# Exploring Complexity of Deliberate Self-Poisoning through Network Analysis

**DOI:** 10.1155/2017/3619721

**Published:** 2017-01-29

**Authors:** Leila R. Kalankesh, Mostafa Farahbakhsh, Rebecca A. Fein, Nazila Moftian, Zahra Nasiry

**Affiliations:** ^1^Tabriz Health Services Management Research Center, Tabriz, Iran; ^2^School of Management and Medical Informatics, Tabriz University of Medical Sciences, Tabriz, Iran; ^3^Tabriz Research Center of Psychiatry and Behavioral Sciences, Tabriz University of Medical Sciences, Tabriz, Iran; ^4^Laboratory Informatics Institute, Smyrna, GA, USA; ^5^National Alliance of Research Associates Programs (NARAP), Hartford, CT, USA

## Abstract

The purpose of this research was to examine the complexity of circumstances that result in deliberate self-poisoning cases. For the purposes of this paper, the cases were patients that presented for care and were admitted to the specialty hospital in Northwest of Iran. The research examined the problems preceding deliberate self-poisoning and the interrelations among them by applying network analysis methods. The network was scored for degrees of centrality and betweenness centrality. Structural analysis of network also was conducted using block modelling. The results showed that family conflicts had the highest score for degree of centrality among women, while the highest score for degree of centrality among men belonged to those dealing with drug addiction. Analysis for degree of betweenness centrality revealed that drug addiction had the highest score among men, whereas the highest score for women on betweenness centrality was related to physical illness. Structural analysis of the network showed differences in role that various problems played in intentional self-poisoning. The findings from this research can be used by public health authorities to create prevention programs that address the problems leading to deliberate self-poisoning.

## 1. Introduction

Incidence rate of suicide attempt in Iran is estimated 91.65 per 100000 (82.2 in men and 115.79 in women) and it is more common in the 15–24 age group [1]. A common method for suicide attempt in both developed and developing countries is deliberate self-poisoning [2–4]. Intentional self-poisoning has also been reported as the most common method of self-harm among patients admitted to the hospital for self-harm incidents [5]. This is also true in Iran [6]. Suicide attempts are not accidents but are deliberate actions taken by individuals that are attempting to free themselves from the pressures that are caused by distressing life circumstances [7]. The importance of investigation on preceding factors and life stressors on attempting suicide has been highlighted [8, 9]. Circumstances that lead to suicide attempts can be classified into two different groups: social circumstances and medical circumstances. Examples of social circumstances are unemployment, divorce, poverty, loss of family members, family problems, and financial problems. Medical circumstances can include mental disorders (depression, bipolar disorder, schizophrenia, etc.) or physical disorders (for example, cancer and multiple sclerosis) [8, 10–12].

While some patients identified family problems as the precursor to the attempted suicide [13–16], others highlighted the link between the attempted suicide and life stressors [17] such as separation, financial problems, work-related problem, unemployment, change of residence place, physical diseases [18], or other health related issues [19] and surgeries [20–22]. Studies of attempting suicides including deliberate self-poisoning have reported factors such as mental disorders, financial and economic problems, unemployment, quarrel with spouse, family conflicts, physical illness and hopelessness, and substance abuse among cases from different parts of Iran [23–25].

It should be noted that suicide and suicide attempts are complex phenomena and often are the result of a complex set of circumstances in the patient's life [26–30] and complex matrix of different interacting variables [16]. Evidence shows the role of combination of risk factors in deliberate self-harm [31]. Comorbidities of multiple factors such as physical and mental problems have been reported among those attempting deliberate self-harm [32]. Some researchers have investigated interconnections among risk factors such as depression, health problems and status of the family support in suicidal ideation [33, 34]. Coexistence of risk factors has been found to increase the probability of attempted suicide compared with existence of only one factor [9, 35]. Understanding interconnection among different factors can be of great importance in formulating suicide prevention programs [36].

Network analysis, originally used for examining the relationship among social entities [37], can be used to perform quantitative analysis of complex systems. Recently this method has been deployed for comparing Diagnostic and Statistical Manual (DSM) and non-DSM symptoms of depression [38] and for explaining comorbidity pattern of mental disorders based on network of psychiatric symptoms derived from DSM-IV [39]. Network analysis method was first applied to examining interconnections between motives for completed suicide attempts using Japanese police statistics [40]. This method allows researchers to form a better understanding of the complexity of factors and problems preceding attempting suicide as it gives us the whole picture of interconnections among all factors contributing to it. Research for this paper examined interconnections among problems preceding deliberate self-poisoning among cases presented and admitted to the hospital (overall and in terms of gender) in the Northwest of Iran through applying network analysis methods.

## 2. Methods

### 2.1. Subjects and Data

A cross sectional study was carried out to assess the problems preceding intentional self-poisoning and their interconnection among cases admitted to the hospital in the Northwest of Iran. The study sample was all cases hospitalized with a diagnosis of deliberate self-poisoning in the second half of the solar year in Iranian calendar. Data were extracted from medical records of all cases (*n* = 357) presented and admitted to a teaching hospital (in Northwest Iran) with a diagnosis of deliberate self-poisoning during 6 months (between September 23, 2013, and March 21, 2014). Patients' records were studied to extract a set of preceding circumstances or problems reported by patients or their family and written in patient history record. List of these variables had been extracted through literature review and had been categorized and validated by the psychiatrist. The data collector was a specialist in field of Health Information Management who had experience of data abstraction from medical records. She was practically trained by psychiatrist on how to extract the data from the patient medical records for this research before starting the data collection.

### 2.2. Generating the Network and Its Measures

A network of the problems preceding intentional self-poisoning was constructed, and interconnections among the problems were depicted as a graph using network analysis methods. The constructed graph contains nodes and the edges between any two connected nodes. Each node represents a distinct problem preceding the intentional self-poisoning and the edge between two nodes represents their cooccurrence. The weight of each edge is determined by frequency of their cooccurrence. GEPHI software [41] was used for constructing and visualizing the network. In addition, centrality measures including degree centrality and betweenness centrality were computed and analyzed for the graph. Degree centrality measure refers to the number of direct connections that an individual node has to other nodes within a network. Increase in number of one factor necessarily may not result in high degree score. In fact, the degree measure of one factor in this paper indicates coexistence of this factor along with other factors. Factors (nodes in the network) with high degree centrality appear to be more important in the network compared with others.

Betweenness centrality measure represents the extent to which a node can act as an intermediary or the most travelled path to other nodes [42]. This measure is of particular interest because it measures the relative importance of a factor by measuring not how well connected it is but instead where it falls between other factors in the network. Any two factors or issues are connected by either a direct path or a path that travels via other factors. Factor with high betweenness score is one that lies along the shortest path relating other factors. Mediation of some of the factors by other factors in suicidal behavior has been reported in the literature [43–46]. The factors with the highest betweenness derive a lot of importance from their position in the network and their removal from the network will disrupt interconnections among other factors. Effective disruption of a network of contributing factors may prevent attempting suicide. It means control or removal of factors with high score of betweenness can be considered as the priority area in prevention strategies for attempted suicide. Example of using degree and betweenness measures in network analysis approach can be found in the literature for investigating the interconnections among contributing factors in other disciplines [40, 47, 48].

### 2.3. Block Modelling of the Network

Structure of the network was examined by the means of block modelling through applying CONCOR* (CONvergence of iterated CORrelations)* algorithm in UCINET (software for block modelling of network). In fact aim of this algorithm is to partition network data by splitting blocks based upon the CONvergence of iterated CORrelations (CONCOR) [49]. The idea of block modelling is to bring out some main features of the network by partitioning the nodes into categories of equivalent nodes. Nodes A and B are structurally equivalent if they relate to other nodes in the same way [50]. In other words, a block model is a representation for clustering objects into groups based upon patterns that occur in the relations among these objects. Two measures of intrablock density and interblock density are used for interpreting structure obtained from block modelling. High intrablock density indicates highly related factors while high interblock density reveals the significant correlation between a pairs of blocks. Average density score of the whole network was considered as the cut-off point for judging on significance of interblock and intrablock density scores. Density of network can be computed through dividing total number of connections in the network by total possible connection in that network [37, 51].

## 3. Results

### 3.1. Basic Characteristics of the Motives

About 57.4 percent of patients (205 cases) were female and 42.6% of them (152 cases) were male. Age of cases ranged between 13 and 84. Average age of the patient was 29.94 (SD = 14). About 18 percent of the cases were under age 20 years, 67% of them aged between 20 and 40 years, and 15 percent of them were over 40 years old. Job categories observed among the patients were housewife (28%), self-employed jobs (15%), middle school and high school pupils (9.34), unemployed (7.2), university students (5%), governmental jobs (2%), soldiers (1.2), and prisoners (0.2). No specified job was mentioned for about 8.5 percent of cases.

The most common substance the cases used for deliberate poisoning was Medicines (77.7%), followed by Pesticides (8%), Tramadol and Methadone (5.5%), Bleach (3.9%), nonspecified substance (3.6%), Petrol (0.8%), and Alcohol (0.5%). In 107 cases, there was only one problem written and reported in their chart. 157 cases had more than one issue written in their records. Overall 579 factors were identified as problems preceding the deliberate self-poisoning and then they were classified into the 6 above-mentioned classes: family problems, health problems, economic problems, romantic problems, educational problems, and others. This categorization was conducted in accordance with the classes of issues recognized in the literature.  Table 1 presents these issues in detail.

### 3.2. Network Visualization and Centrality Measures

**Figure 1:**
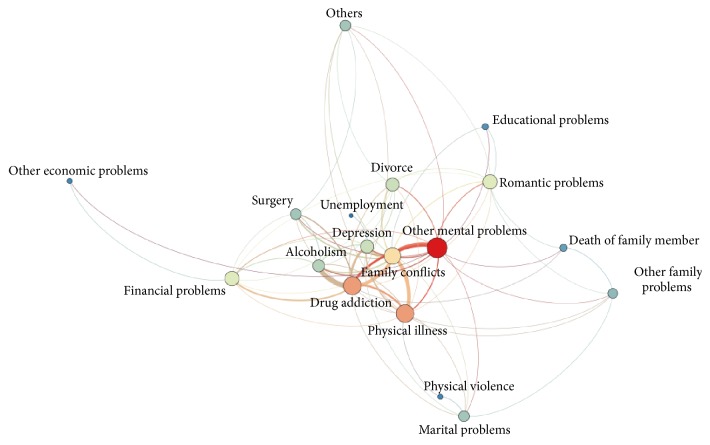
Network of issues preceding the deliberate self-poisoning.

The network constructed for visualizing the interconnection of the issues and circumstances preceding the intentional self-poisoning are depicted in Figure 1. Moreover, the centrality measures for each class of the problems are provided in Table 1.

### 3.3. Degree Centrality Measures of the Network

As Table 1 shows the class of health problems has the greatest degree (0.45) followed by the family problems (degree centrality of 0.37), meaning that these two classes of issues had the first and second highest number of connections with other categories. Among subclasses of family problems, family conflicts had the highest degree centrality (0.178) and among subcategories of health issues, drug addiction had the highest degree centrality (0.173) followed by other mental problems (0.125) and physical illness (0.102). In fact these are the issues highly connected with other problems.

By looking at degree centrality scores in terms of gender, it is noticeable that the issues in the class of family problems are more connected to others in women (degree centrality of 0.416) compared with men (0.302). In subcategories of family problems, the most highly connected issue is family conflicts among both men and women. However, degree centrality of this issue among women (0.241) is twice as much the score among men (0.126).

Health problems have approximately equal number of connections with other factors in both men (degree centrality of 0.45) and women (degree centrality of 0.448). In subclasses of health problems, three most highly connected factors to others among women are other mental problems (degree centrality of 0.152), physical illness (degree centrality of 0.131), and depression (degree centrality of 0.103) while these factors among men include drug addiction (degree centrality of 0.258), alcoholism (degree centrality of 0.147), and other mental problems (degree centrality of 0.107).

### 3.4. Betweenness Centrality Measures of the Network

As it can be seen from Table 1, the betweenness centrality measure was found to be equal for both health and family problems. Looking at subcategories of each class, the four highest betweenness centrality scores belong to other mental problems (0.185), depression (0.122) physical illness (0.118), and drug addiction (0.07), respectively.

Betweenness centrality score for family problems was found to be higher among women (0.167) compared with men (0.05). Moreover, when it comes to the subcategories of this class, family conflict had higher score of betweenness centrality among women (0.147) compared with men (0.09).

Higher score of betweenness centrality score was revealed for health problems among men (0.65) compared with women (0.25). In subcategories of health problems, betweenness scores of physical illness were found to be higher among women (0.172) than men (0.096), while the score of depression was found to be higher for men (0.135) compared with women (0.005).

Similarly the betweenness scores of drug addiction (0.196) and other mental problems (0.186) were higher for men compared with those among women.

### 3.5. Structure of Total Network

**Figure 2:**
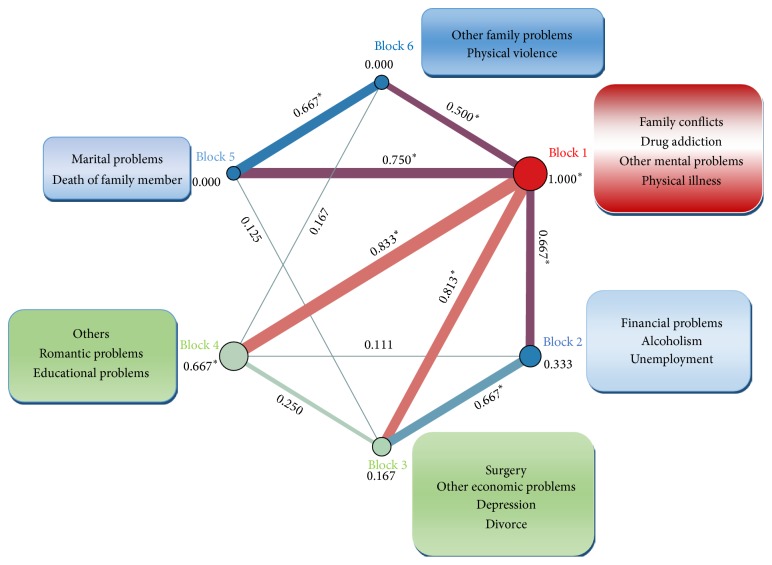
Block modelling in the network of problems preceding deliberate self-poisoning. ^*∗*^Density scores higher than the score of whole network.

As Figure 2 illustrates, overall block modelling has categorized problems preceding the deliberate self-poisoning into 6 blocks. Numbers under and between the nodes refer to the intrablock and the interblock density scores, respectively. The most important problems characterized by the degree centrality score have been clustered into block 1 which has the highest intrablock density. Moreover, this block has connection with all other blocks in the network (with the interblock density score higher than the score of whole network (1 > 0.44)).

### 3.6. Structure of the Network among Women

**Figure 3:**
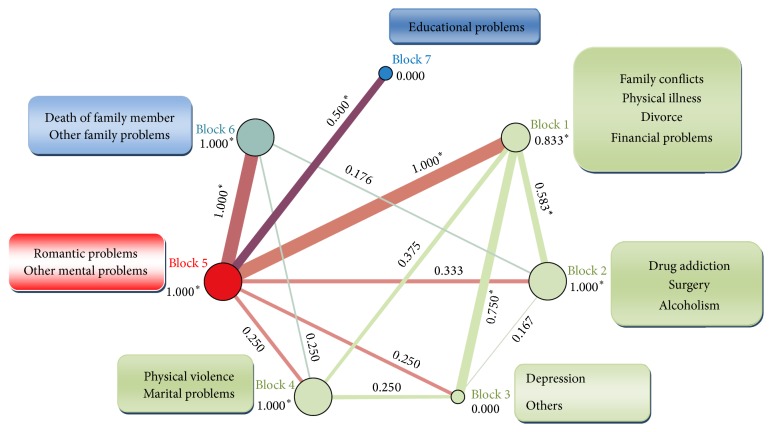
Block modelling in the network of problems preceding deliberate self-poisoning among women. ^*∗*^Density scores higher than the score of whole network.

The structure of problems proceeding the deliberate self-poisoning among women has been depicted in Figure 3. As it can be seen from this figure, both family conflicts (as the first important problem in terms of the degree centrality score) and physical illness (as the third important problem in terms of degree centrality) have been clustered into block 1 while depression and other mental problems have been classified in block 3 and block 5, respectively. The highest score of interblock density can be observed between block 1 and block 5 and between block 5 and block 6, followed by interblock density between block 1 and block 3.

**Figure 4:**
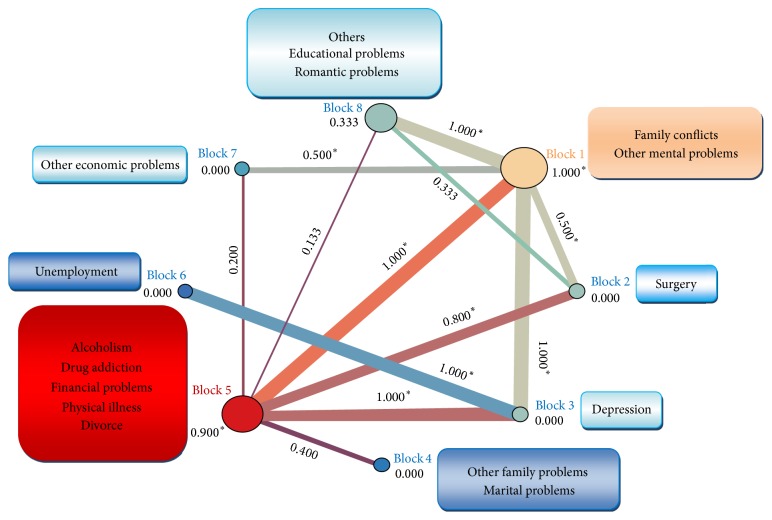
Block modelling in the network of problems preceding deliberate self-poisoning among men. ^*∗*^Density scores higher than the score of whole network.

As Figure 4 shows the block modelling algorithm has clustered the most important problems identified among men into 4 separate blocks. Block 1 and block 5 have the intrablock density higher than the whole network. Drug addiction as the most important problem among men has been located in block 5 while both family conflict (as the second important problem) and other mental problems (as the third important problem) have been clustered together into block 1. Depression (as the fourth important issue) has been completely isolated from other issues and has been placed in block 3.

## 4. Discussion

This study revealed the pattern of problems preceding deliberate self-poisoning in terms of their importance, their interconnection, and their role. In terms of the main classes of factors, health problems are the most highly interconnected factor. In fact this main category has connection with every other category of factors in the network. This is in consistent with the results from Japanese study [40]. Inside the category of health problems three most interconnected issues are drug addiction, other mental problems, and physical illnesses. Cooccurrence of drug addiction with severe mental health problems has been highlighted in the literature [52]. Moreover, emphasis on importance of employment intervention on substance abuse treatment reveals interrelation between drug addiction and unemployment [53]. Interconnection between family conflicts or problems with drug abuse has been documented in the literature as well [54].

The second most interconnected issue is family problems. This differs from finding of Japanese study in which economic and livelihood problems have been found to be as the second interconnected issues in the network [40]. Previous studies have reported family conflicts among the common and important factors contributing to the intentional self-harm and attempting suicide in Iran and other countries [16, 55–57].

Overall three highest scores of betweenness centrality belong to other mental problems (including character disorder, schizophrenia, and bipolar disorder), depression, and physical illness, respectively. It means mental problems and physical illness have important role in interrelations among other factors. This implies that disrupting network through these factors can be very effective in preventing attempting suicide.

Based on analysis of the network in terms of gender, drug addiction was found to be the most highly connected issue in the network among men. Moreover, this issue in males played as an intermediary issue among other factors. This was indicative of its centrality and importance in the deliberate self-poisoning among men. According to the recent meta-analysis conducted on prevalence and pattern of drug use among Iranian adolescents, males are in considerably greater risk of drug use compared with females [58]. This finding was completely different from the result found in the study conducted on the completed suicide cases in Japan, in which drug abuse was not such an important factor among either men or women. This might be attributed to some extent to the differences in nature of completed suicide and the attempting suicide. Furthermore, the issue of drug addiction might be a problem specific to Iranian society with its own related individual or social problems; the Iranian community considers addiction the third biggest threat to their society after the inflation and unemployment [59]. Comorbidity of drug addiction with personality disorders has been reported in a study carried out in west part of Iran [60]. This implied that any successful intervention on drug addiction issues can break down the network and may result in the prevention of deliberate self-poisoning. This means intervention strategies for preventing deliberate self-poisoning among men should give priority to the issue of drug addiction as the main factor in attempted self-poisoning among men. Boys must be the main target of preventive programs in this aspect. Prevention initiatives including life skill training and parenting skill programs with focus on adolescents have been suggested in the literature since it is believed that the onset of substance use and abuse is during adolescents [61, 62].

When it comes to women, family conflicts were found to be the most connected with and the most critical issue preceding intentional self-poisoning. This finding was in line with the finding of a study from Karachi in which women had been found to attempt suicide more compared with men due to the family conflicts [63]. Families in Iranian community are witnessing a change in which interplay between tradition and modernity is happening [64]. This may influence women members of the family even more. Moreover, family member may belong to different generations who could not understand each other. This may result in family conflict. Evidence shows that some of the factors are gender-specific in attempting suicide. For example, financial problems and Alcohol use disorders have been reported as a risk factor specific to the males while weakness of connection with family has been highlighted to be specific to the females [65, 66].

Overall block modelling of the network was indicative of similar roles played by family conflicts, drug addiction, other mental problems, and physical illness in deliberate self-poisoning. These four issues have been clustered into block 1 with highest score of intrablock density. Meanwhile, these are issues with the highest score of degree centrality in the network. Issues located in this block constitute about 51% of all problems extracted. This implies that 51 percent of the problems have similar roles in deliberate self-poisoning. This reflects the importance of factors in this block. Financial problems, alcoholism, and unemployment have been clustered into block 2. Overall 8.5 percent of the problems are included in this cluster. Scores of interblock density of block 1 with other blocks imply the reinforcing effect of factors in this block on others.

Among men, the role of other mental problems and family conflicts was found to be similar in deliberate self-poisoning while the role of drug addiction was found to be different from the family conflicts and other mental problems. This factor has been clustered inside block 5 which includes alcoholism, physical illness, divorce, and financial problems. This can reflect the similar roles played with these five factors in deliberate self-poisoning. Block 1 and block 5 are the distinguished blocks of the model as their intrablock and interblock density measure is high. This reveals the high importance of factors clustered inside these blocks and their interrelations. Depression was revealed to have totally different role among men as it has been clustered in completely separate block. Moreover, high interblock density between block 3 and block 6 reveals an important relation between depression and unemployment in attempting self-poisoning among men. There is evidence from longitudinal study suggesting that unemployment predates depression among men [67]. Depression as one of the main mental diseases can also be referred to a pathway between unemployment and attempting suicide [68]. Since depression has the third highest score of betweenness among men, it should be given the priority in planning prevention strategies for attempting suicide among men. This is in accordance with the fact that major depression provides the background for most of the suicide attempts [69].

Family conflicts and physical illness were found to play similar role in intentional self-poisoning among women. Depression was found to have totally different role from other issues. This may reflect distinct function of different problems among those attempting intentional self-poisoning as well as the function of gender in these differences. Important role of some factors in suicidal behaviors among one gender compared with another has been highlighted in the related literature [65]. As it is evident, factors in block 1 have important relations with the depression among women. This result is consistent with the result of study which reported that family conflicts trigger depression among women [70]. Our findings imply the interrelation between depression and physical illness as well. This is contrary to the result of study in which physical illness was not reported as the trigger for depression [70].

This research may inform public health authorities about the important problems preceding the deliberate self-poisoning and interrelations among those issues in order to establish appropriate prevention strategies. Some consider suicide as a proxy measure for unmet needs among vulnerable individuals [71] and the suicide attempt can be as “a cry for help.” Therefore, prevention strategies should be based on providing the vulnerable individuals with the services or skills they need to cope with their problems or to resolve them. The prevention strategies and programs should also be tailored for vulnerable individuals of different genders specifically. Evidence suggests that it is required to put in place a combination of gender-specific preventive approaches in order to address different risk factors and problems preceding suicidal behaviors [72].

Examples of such prevention strategies would be providing free community-based services for family counselling, training living skills, problem solving, effective communication with family members and appropriate parenting mechanism to address family conflicts [73, 74]. It is also required to make available case management services depression in primary care in the population level and to ensure active follow up of depressed patients [75] or to provide suicide screening program for those suffered from mental disorders.

## 5. Conclusion

Network analysis methods provide useful tools to quantify and better understand a network of interconnections among issues preceding the deliberate self-poisoning. Through information provided from this analysis, the high priority issues contributing to the deliberate self-poisoning can be characterized in order to guide the priorities for intervention programs. Findings from such analyses may also inform the public health authorities for making evidence-based prevention programs in which network of important factors and issues contributing to the deliberate self-poisoning are addressed. A combination of preventive programs can be implemented to influence the network of issues behind the deliberate self-poisoning. Such prevention programs may have much more effective influence than prevention programs that target only one single factor. However, those factors or problems that have high degree and betweenness and have been clustered in separate clusters should be considered in prevention programs.

## Figures and Tables

**Table 1:** Pattern of problems preceding deliberate self-poisoning and their related centrality measures in the network.

Categories	Number (%)			Centrality measures					
	Total	Male	Female	Total		Male		Female	
				Degree	Betweenness	Degree	Betweenness	Degree	Betweenness
Health problems	276 (47.6)	146 (0.55)	130 (0.41)	0.45	0.183	0.448	0.65	0.449	0.25
Physical illness	49 (8.4)	13 (4.9)	36 (11.4)^*∗*^	0.102	0.118	0.074	0.096	0.131	0.172
Depression	49 (8.4)	15 (5.6)	34 (10.8)^*∗*^	0.097	0.122	0.086	0.135	0.103	0.005
Alcoholism	25 (4.3)	23 (8.6)^*∗*^	2 (0.6)	0.087	0.003	0.147	0.009	0.017	0
Drug addiction	69 (11.9)	59 (22.2)^*∗*^	10 (3.1)	0.173	0.070	0.258	0.196	0.059	0.074
Other mental problems^*∗∗*^	67 (11.5)	30 (11.3)	37 (11.7)	0.125	0.185	0.107	0.186	0.152	0.119
Surgery	17 (2.9)	6 (2.2)	11 (3.5)	0.049	0.002	0.034	0.007	0.055	0.010
Family problems	212 (36.6)	66 (0.25)	146 (0.47)	0.37	0.183	0.302	0.05	0.416	0
Family conflicts	156 (26.9)	56 (21.1)	100 (31.8)^*∗*^	0.178	0.057	0.126	0.090	0.241	0.147
Marital problems	22 (3.7)	4 (1.5)	18 (5.7)^*∗*^	0.023	0.036	0.009	0	0.041	0.045
Divorce	15 (2.5)	4 (1.5)	11 (3.5)	0.045	0.008	0.034	0	0.069	0.029
Death of family member	5 (0.8)	0 (0)	5 (1.5)^*∗*^	0.005	0	—	—	0.014	0.010
Physical violence	3 (0.5)	0 (0)	3 (0.9)	0.003	0	—	—	0.007	0
Other family problems	11 (1.8)	2 (0.7)	9 (2.8)	0.011	0.007	0.006	0	0.014	0.011
Economic problems	29 (5)	26 (0.098)	3 (0.01)	0.088	0	0.164	0	0.026	0
Financial problems	23 (3.9)	20 (7.5)^*∗*^	3 (0.9)	0.047	0.044	0.067	0.057	0.017	0.003
Unemployment	2 (0.3)	2 (0.7)	0 (0)	0.005	0	0.003	0	—	—
Other economic problems	4 (0.6)	4 (1.5)^*∗*^	0 (0)	0.003	0	0.006	0	—	—
Romantic problems	16 (2.7)	5 (1.8)	11 (3.5)	0.024	0.042	0.017	0	0.065	0
Educational problems	5 (0.8)	3 (1.1)	2 (0.6)	0.005	0	0.009	0	0.003	0
Others^**∗∗∗**^	41 (7)	19 (7.1)	22 (7)	0.018	0.002	0.018	0.005	0.021	0

Total	579	265	314						

*∗* refers to the statistical difference between men and women. ^*∗∗*^Category of “other mental problems” includes personality disorder, bipolar disorder, and schizophrenia.

^*∗∗∗*^Category of “others” includes work place conflicts, failure in sport competition, conflict with friends, heavy conviction, and escape from military service.

## Additional Points

This research was limited to the data reported by patients or their family on motives behind self-poisoning and recorded in their medical charts. Therefore, there might be a possibility of under reporting some of the issues by them. Data obtained from clinical and psychiatric assessment of patients by clinicians might result in different findings as the statistics on distribution of motives behind deliberate self-poisoning implies. Conducting this analysis on large number of patients was not feasible in this project due to resource limitation. Applying this methodology on large number of data in the future research can shed some light on possibility of sample size effect on the analysis result. Separate network analysis for interrelations of problem among those with mental disorders and without mental disorders can clarify the role of different issues when they are cooccurred with mental disorders.
